# Assessment of the Impact of Different Treatments on the Technological and Antifungal Properties of Papyrus (*Cyperus Papyrus* L.) Sheets

**DOI:** 10.3390/ma12040620

**Published:** 2019-02-19

**Authors:** Ayman S. Taha, Mohamed Z. M. Salem, Wael A. A. Abo Elgat, Hayssam M. Ali, Ashraf A. Hatamleh, Eslam M. Abdel-Salam

**Affiliations:** 1 Conservation Department, Faculty of Archaeology, Aswan University, Aswan 81528, Egypt; aymansalahtaha82@yahoo.com; 2 Forestry and Wood Technology Department, Faculty of Agriculture (EL-Shatby), Alexandria University, Alexandria 21526, Egypt; zidan_forest@yahoo.com; 3 High Institute of Tourism, Hotel Management and Restoration, Abu Qir, Alexandria 21526, Egypt; watsat20@yahoo.com; 4 Botany and Microbiology Department, College of Science, King Saud University, P.O. Box 2455, Riyadh 11451, Saudi Arabia; ahatamleh@ksu.edu.sa (A.A.H.); 436108002@student.ksu.edu.sa (E.M.A.-S.); 5 Timber Trees Research Department, Sabahia Horticulture Research Station, Horticulture Research Institute, Agriculture Research Center, Alexandria 21526, Egypt

**Keywords:** papyrus, mechanical, biological properties, antifungal activity

## Abstract

In the present work, sheets of Papyrus (*Cyperus papyrus* L.), manufactured by lamination from strips pre-treated with different treatments, were evaluated for their technological and fungal infestation properties (*Aspergillus flavus* AFl375, *A. niger* Ani245 and *Colletotrichum gloeosporioides* Cgl311). The results showed that the highest values of tensile strength, tear strength, burst index and double-fold number were observed in papyrus sheets produced from strips treated with nano-cellulose (0.25%), dimethyl sulfoxide (DMSO 10%), Tylose (0.25%) and nano-cellulose (0.5%), with values of 98.90 N·m/g, 2343.67 mN·m^2^/g, 1162 kpa·m^2^/g and 8.33, respectively. The percentage of brightness ranged from 49.7% (strips treated with KOH 2% + 100 mL NaClO) to 9.6% (strips treated with *Eucalyptus camaldulensis* bark extract 2%), while the percentage of darkness ranged from 99.86% (strips treated with *Salix babylonica* leaf extract 2% or *E. camaldulensis* bark extract 0.5%) to 67.26% (strips treated with NaOH (2%) + 100 mL NaClO). From the SEM examination, sheets produced from treated strips with extracts from *P. rigida* and *E. camaldulensis* or *S. babylonica* showed no growths of *A. flavus* and *C. gloeosporioides*. Additionally, other pre-treatments, such as Nano-cellulose+Tylose 0.5% (1:1 *v*/*v*) and Tylose 0.5%, were also found to have no growth of *A. niger*. In conclusion, strips pre-treated with nanomaterials and extracts were enhanced in terms of the technological and antifungal properties of produced Papyrus sheets, respectively.

## 1. Introduction

*Cyperus papyrus* L. (commonly known as papyrus), a remarkable emergent macrophyte, belongs to the family of Cyperaceae, reaching 4 m in height and growing along the banks of the Nile and was used by Egyptians as a flexible writing support of vegetable origin [1]. Papyrus stems, bundled together, were used by the Egyptians for boat making and they also wove the papyrus fibres into water resistant ropes, mats, baskets and tables [2]. It has been used over millennia, such as in the manufacture of the first paper by the ancient Egyptians [3,4,5] and for mummy wrappings [6]. Recently, Papyrus stems have been studied in relation to their utilization in furniture, mats, making fences, roofing, matting baskets and biofuel or briquettes [5,7,8].

Papyrus stems can be harvested by hand and stacked on a rhizome mat for partial air-drying, before Culms, the main aboveground vegetative structure, form the Papyrus as floating monotypic stands. Culms are topped by an umbel, consisting of numerous cylindrical rays and flattened, leaf-like, bracteoles [7]. 

Papyrus made from the pith, the inner white part of the rectangular stem, after being peeled and having the green outer rind removed, is flexible and light and it is composed mainly of cellulose (53.29–62.04%) and lignin (22.42–32.77%) [9]. The pith is then sliced longitudinally to produce strips, which are laid side by side to form one layer. More strips are then placed on top, at right angles, to form a second layer [3]. After being dried, the sheet is polished with some rounded object, possibly a stone, seashell or round hardwood [10]. This technique was also used with the stems of *Tetrapanax papyrifera* [11]. 

Different material types, such as cellulose ethers, the sodium chloride of carboxymethylcellulose (CMC), Chitosan and methylcellulose (MC), have been used to improve the mechanical properties, consolidation, strength and scratch resistance of Papyrus sheets, as well as their possible resistance to biological attack from the manufactured objects [12,13,14,15,16,17].

This study aimed to evaluate the effect of various additives on the technological properties and the fungal resistance of the papyrus during manufacturing, in an attempt to improve the natural properties of papyrus sheets.

## 2. Materials and Methods 

### 2.1. Chemicals Used

Different chemical compounds, such as nano-cellulose (Sigma-Aldrich, Darmstadt, Germany), Tylose (Methyl hydroxyethyl cellulose) (Sigma-Aldrich, Darmstadt, Germany), dimethyl sulfoxide (Sigma-Aldrich, Darmstadt, Germany), as well as NaOH, KOH and NaClO (Loba Chemie Pvt. Ltd., laboratory reagents & fine chemicals, Mumbai, India), were used.

### 2.2. Preparation of Papyrus strips

**Figure 1:**
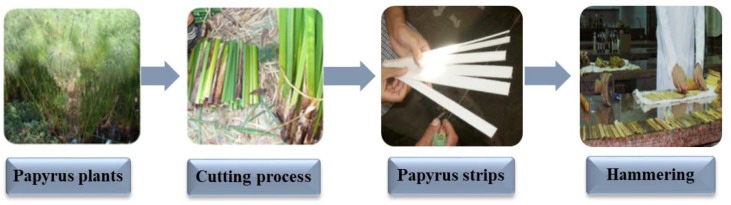
Preparation of Papyrus strips from the plants.

Papyrus stems were cut and collected from Al-Qaramous, Abu-Kbir, Alshrqia, Egypt, in August 2017. Stems were peeled and the green outer rind was removed, then the pith (composed mainly of cellulose and lignin) was cut into strips and hammered (Figure 1) [11].

Briefly, for 3–7 h and after harvesting the plant, the sheets were made, which involves the easy process of peeling off the outer green bark and avoiding sticking it to the white inner pith. The crust or outer layer was removed using a sharp knife to obtain good slices. The sticky fibrous inner pith was cut lengthwise into thin strips of about 40 cm (16 in.) in length. The strips were then placed side by side on a hard surface, with their edges slightly overlapping and another layer of strips was laid on top at a right angle [18].

For hammering, whole strips were beaten or pressed together to form a homogeneous sheet, which is then dried. Aided by the natural sap contained in the plant, the pressure applied during this procedure fused the cellulose in each layer together physically and chemically. Mechanical rotating pressing rollers were used to squeeze these strips to facilitate better mechanical wet-bonding, just before they are manufactured [4].

For drying, however, we did not have details on the Egyptian technical process. The two layers were hammered together, mashing the layers into a single sheet and then dried under pressure, allowing two layers of papyrus pith strips to adhere to one another and improve the surface regularity, making the sheet smoother under the scribe’s brush [19,20]. All the manufacturing processes were conducted in laboratory conditions, where the shadow area is maintained to allow the stalks to remain wet; in case they exposed to drying conditions, the stalks are rewet [3,4,5].

### 2.3. Pre-Treatments of Papyrus strips

**Figure 2:**
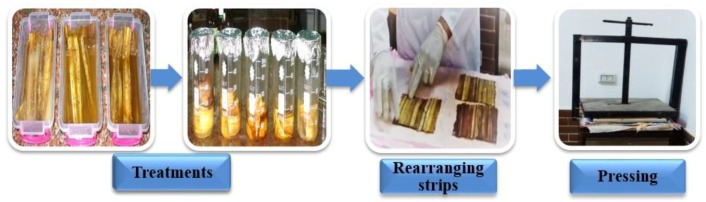
Papyrus strips treated with different treatments and sheet formation by lamination, then cold pressed.

**Figure 3:**
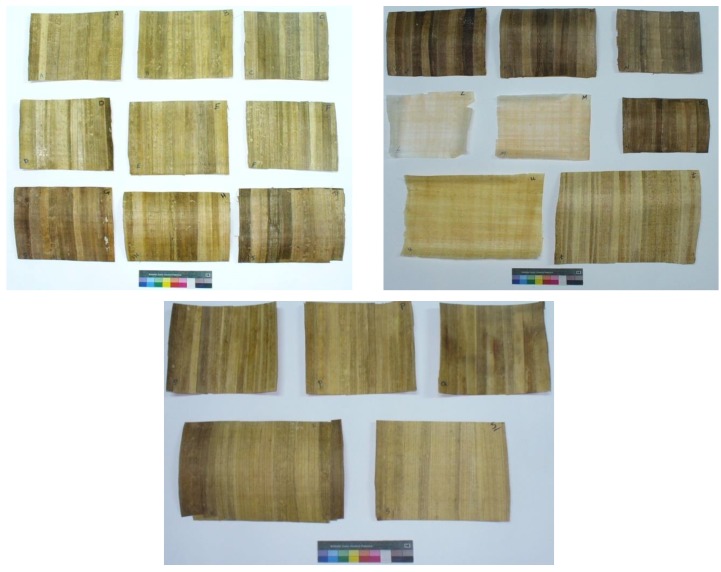
Manufactured papyrus sheets.

Strips were treated with 24 treatments (Table 1). Each treatment was carried out in triplicate. The treated strips were joined together and rearranged by lamination, then pressed down to form a sheet (Figure 2), as described above. This method was used in a manufacturing process similar to archaeological papyrus. The manufactured papyrus sheets are presented in Figure 3. 

#### Preparation of Extracts and GC/MS Analysis

Extracts were prepared according to the method described by EL-Hefny et al. [21], where 50 g from each air-dried material of *Eucalyptus camaldulensis* bark, *Pinus rigida* heartwood and *Salix babylonica* leaf were ground to fine powder (40–60 mesh) and soaked in 150 mL of methanol for one week, then filtrated through filter paper (Wattman No. 1). The extracts were concentrated, after evaporating the solvent at 40 °C using a rotary evaporator and stored at 4 °C until analysis. The extracts were prepared at the concentrations of 0.5%, 1% and 2% by dissolving the extracts in dimethyl sulfoxide (DMSO 10%).

The suggested chemical composition of methanol extracts was analysed by Trace gas chromatography (GC) using an Ultra-ISQ Mass Spectrometer (Thermo Scientific, Austin, TX, USA), with a direct capillary column TG-5MS (30 m × 0.25 mm × 0.25 μm film thickness) apparatus from the Atomic and Molecular Physics Unit, Experimental Nuclear Physics Department, Nuclear Research Centre, Egyptian Atomic Energy Authority, Inshas, Cairo, Egypt. The column oven temperature programs and the properties of sample injection can be found in previously published work [22].

### 2.4. Testing of Papyrus Sheets

**Figure 4:**
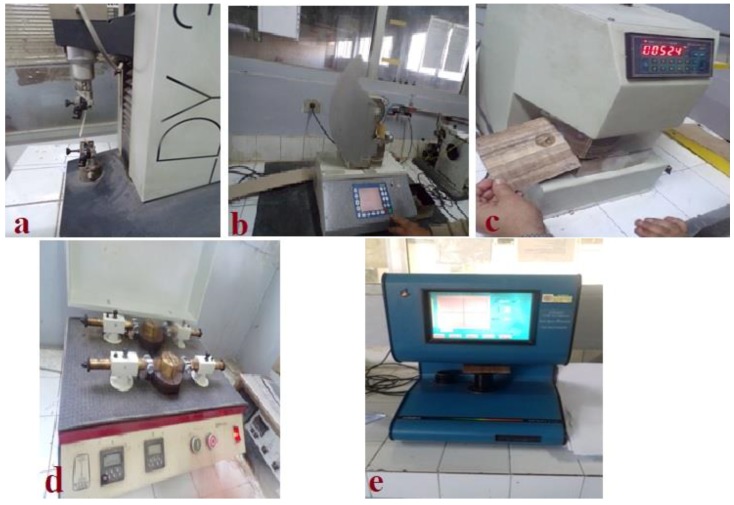
Apparatuses for measuring the mechanical and physical properties of the manufactured papyrus sheets. (**a**) Tensile testing machine; (**b**) Tear testing machine; (**c**) Burst strength tester BS 20T; (**d**) Double-fold number DBF 6-60; (**e**) Spectrophotometer Color.

The tensile strength was tested by a tensile testing machine, model DY 30 (Adamel Lhomary-France) (Figure 4a) [23]. The tear testing machine (model 53984, FRANK-PTI GMBH, Germany) (Figure 4b) was used for determining the tear strength [24]. A burst tester machine (model BS 20T, Iorgen Witte–Germany), shown in Figure 4c, was used as a multi-directional tensile tester to identify failure in the direction of least resistance [25]. A double-fold number tester, model DFP (6–60), manufactured by Kogel Leipzig (Leipzig, Germany) and shown in Figure 4d, was used to determine the total number of folding on a digital screen [26]. The optical properties were measured using Spectrophotometer Color Touch (model, Iso, Technidyne Corporation, New Albany, OH, USA).

### 2.5. Antifungal Activity In Vitro

Discs of sheet samples, with the dimension of 0.5 × 0.5 cm^2^, from the 24 manufactured sheets were prepared. All the samples were subjected to the fungal exposure, following the published works [27,28]. Sheet samples were exposed to fungal infestation using three moulds (*Aspergillus flavus* AFl375, *A. niger* Ani245 and *Colletotrichum gloeosporioides* Cgl311) and these fungal strains were isolated from infected ancient tissue/textile or archaeological manuscripts, organic materials (wood, parchment), molecularly identified using DNA sequences of partial ITS gene and accessioned the numbers in GenBank, MH355958, MH355955 and MH355957, respectively. Sheet samples were inoculated with a 5 mm disc diameter and 15-day-old PDA culture from each fungus in petri dishes and incubated for 14 days at 25 ± 1 °C. For each fungus, the growth on disc (mm) and inhibition zone (mm), after 7 and 14 days from inoculation, were recorded.

### 2.6. Scanning Electron Microscopy

Fungal infestations over the papyrus sheets manufactured with 24 treatments were examined using a Scanning Electron Microscope (SEM, JFC-1100E Ion sputtering device model JSM–5300, JEOL Co., Tokyo, Japan) at 8 kV. 

The criteria for choosing the samples subjected to SEM examination are as follows:

1. Some treatments reflect the effect of fungus on the fibres without any treatments and thus show the intensity of fungal growth and degree of resultant damage.
2. Some of them reflect the highest concentration applied to the materials and thus clarify its effects on the growth of fungi.
3. Shows the effect of the treatment solutions on the precise anatomy of the fibres treated with alkaline materials, such as sodium hydroxide, potassium hydroxide and bleach and illustrates the effect of these materials on the growth of fungi.
4. Illustrates the effect of the different manufacturing stages (such as a hammering phase) on the degree of damage in the anatomical structure and the fungal growth on the manufactured samples.
5. Shows the effect of the mixture treatments on the physical and biological properties of the manufactured papyrus sheets.

### 2.7. Statistical Analysis

Data of the mechanical and physical properties of manufactured papyrus sheets were statistically analysed with ANOVA using an SAS system [29]. Comparisons of the means were recorded using LSD_0.05_.

## 3. Results and Discussions

### 3.1. Mechanical and Optical Properties 

The mechanical and optical properties of the manufactured papyrus sheets, as affected by 24 treatments, are presented in Table 2. All the properties are affected significantly by the treatments. The highest tensile strength (TS) values are observed with the treatments of nano-cell 0.5%, nano-cell 0.25% and STW+NaClO, with 98.66, 98.90 and 98.63 N·m/g, while the lowest values were 43.33, 55.16, 60.40 N·m/g, where the papyrus sheets were treated with the treatments of KOH 2% + 100 mL NaClO, NaOH (2%) + 100 mL NaClO and CPS, respectively. The highest tear strength (TS) values were 2343.67, 2233 and 2241.33 mN·m^2^/g for the papyrus sheets treated with DMSO 10%, Tyl 0.5% and Tyl 0.25%, while the lowest values were found with the treatments of KOH 2% + 100 mL NaClO, NaOH (2%) + 100 mL NaClO and CPS, with 910.67, 871 and 807.67 mN·m^2^/g, respectively.

The burst index (BI) showed the highest values of 833.33, 1162, 795.33, 794.67 and 817 kPa·m^2^/g, with manufactured papyrus sheets pre-treated with Tyl 0.5%, Tyl 0.25%, nano-cell + Tyl 0.5% (1:1 *v*/*v*), nano-cell + Tyl 0.25% (1:1 *v*/*v*) and nano-cell 0.5%, respectively. Papyrus strips pre-treated with KOH 2%+100 mL NaClO, NaOH (2%) + 100 mL NaClO, STW + NaClO, CP+2%KOH + NaClO and CPS, showed the lowest BI in the manufactured papyrus sheets, with values of 196.67, 312.67, 320, 365.33 and 388 kPa·m^2^/g, respectively. Comparing these results with other published works on BI (kPa·m^2^/g), paper manufactured with the pulp of alfa, bamboo, giant reed, Miscanthus, reed cannery, switch grass and Napier grass had the BI values of 1.30 [30], 2.02 [31], 0.50 [32], 1.23 [33], 4.00 [34], 5.30 [35] and 4.98 [36], respectively. The greatest variations in BI among the manufactured papyrus sheets and paper sheets from other lignocellulosic materials resulted from the papyrus sheets manufactured with two thick layers as laminations.

The highest double-fold numbers were observed in the sheets manufactured with pre-treated strips with Nano-cell 0.5%, STW + NaClO, CP + 2% KOH + NaClO and SUP, with values of 8.33, 7.33, 6.66 and 6.33, respectively. On the other hand, the lowest values were reported with treatments of KOH 2% + 100 mL NaClO (1.66), NaOH (2%) + 100 mL NaClO (2.33) and CPS (2.66).

The highest brightness percentage of the manufactured papyrus sheets were 49.7, 40.1, 27.86 and 25.43% with the treatments of KOH 2% + 100 mL NaClO, NaOH (2%) + 100 mL NaClO, STW + NaClO and CPS, respectively, while the lowest values of 9.6, 11.63, 11.53, 11.56 and 11.3% were observed with the treatments of EuBEx 2%, EuBEx 1%, EuBEx 0.5%, DMSO 10% and SUP, respectively.

Comparing these results with those of other lignocellulosic materials, the percentages of brightness of the manufactured papers were 14.1% (palm midribs), 37.3% (wheat straw) and 18.1% *Juniperus procera* wood [37]; 47.30% (Alfa) [30], 39.92% (Bamboo) [31], 22.8% (Giant reed) [32], 30.1% (Switch grass) [35] and 74.6% (Napier grass) [36].

The highest darkness percentages of the papyrus sheets were 99.76, 99.86, 99.76, 99.7, 99.8, 99.86 and 99.8% for the strips pre-treated with PPWEx 0.5%, SLEx 2%, SLEx 0.5%, EuBEx 2%, EuBEx 1%, EuBEx 0.5% and STW + NaClO, respectively, while the lowest values were observed with treatments of KOH 2% + 100 mL NaClO, NaOH (2%) + 100 mL NaClO and CP + 2%KOH + NaClO, with values of 62.53, 67.26 and 72.73%, respectively. 

The strips pre-treated with PPWEx 1% and Tyl 0.5% showed the highest basis weight values of 220 and 368.88 g/m^2^, respectively, while the lowest were obtained from the treatments of KOH 2% + 100 mL NaClO and NaOH (2%) + 100 mL NaClO, with values of 82.25 and 94.25 g/m^2^, respectively. Handmade paperboard sheets from old newsprint (fibres of rice straw) were found to have a 120 g/m^2^ basis weight [37].

### 3.2. Visual Observations of the Antifungal Activity of Treated Papyrus Sheets

**Figure 5:**
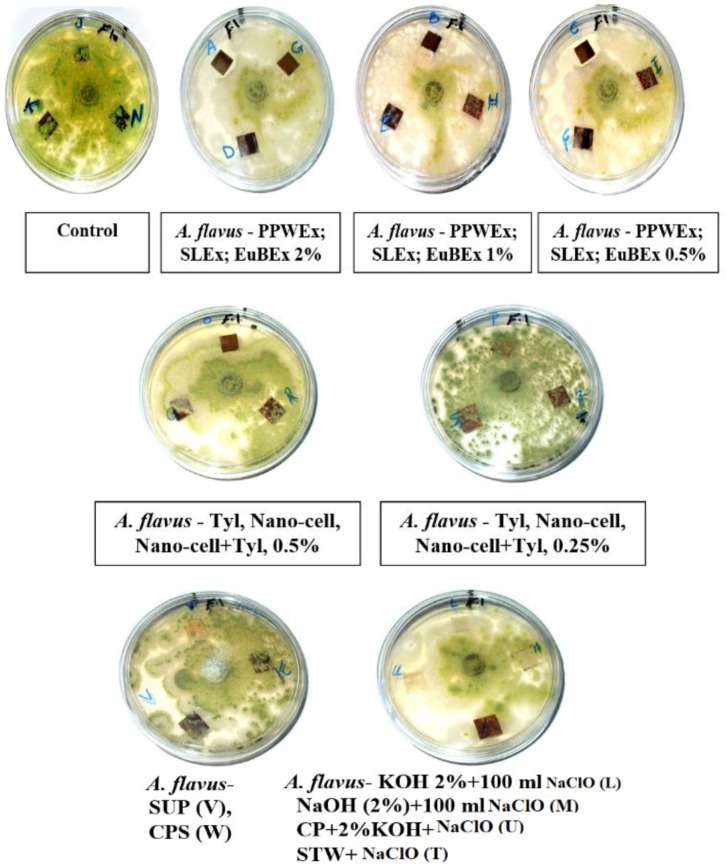
Antifungal activity of papyrus sheets against the growth of *A. flavus*.

**Figure 6:**
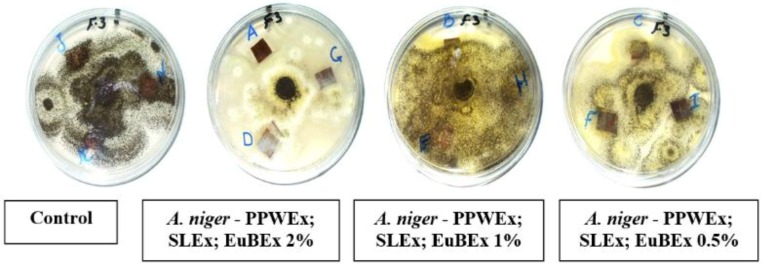

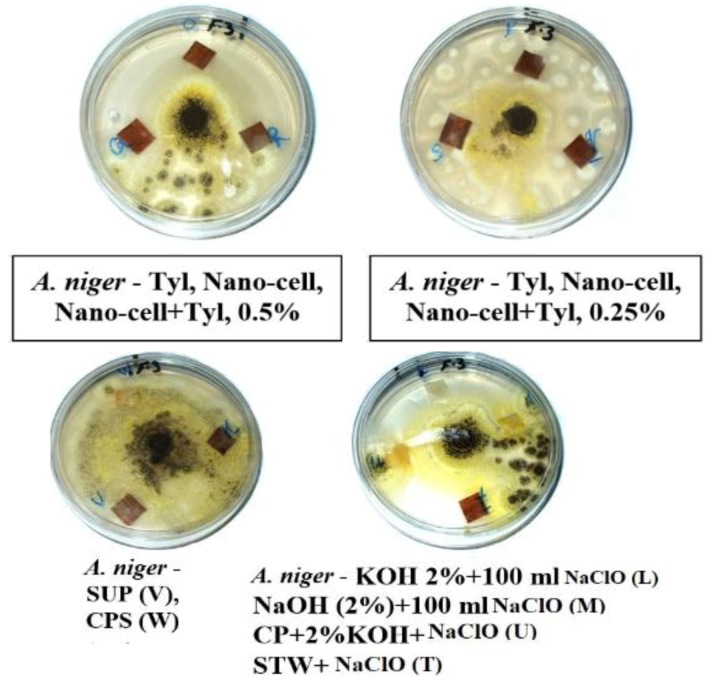
Antifungal activity of papyrus sheets against the growth of *A. niger*.

**Figure 7:**
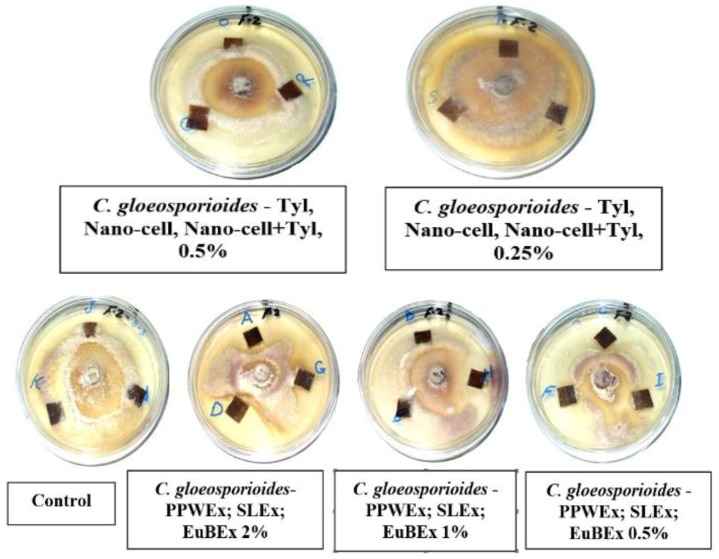

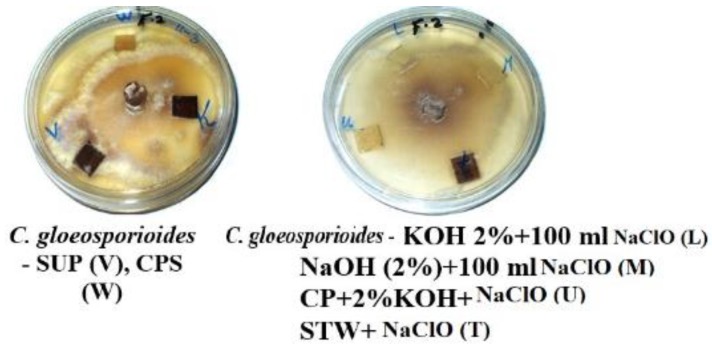
Antifungal activity of papyrus sheets against the growth of *C. gloeosporioides*.

Figure 5, Figure 6 and Figure 7 present the antifungal activity of manufactured sheets against three moulds (*Aspergillus flavus*, *A. niger* and *Colletotrichum gloeosporioides*). The inhibition zones (mm) as well as the growth on the discs are presented in Table 3. Compared to the control treatments, after 14 days from the incubation, nearly no growth of *A. flavus* was found in the Papyrus discs manufactured with PPWEx 2%, PPWEx 1%, PPWEx 0.5%, EuBEx 2%, KOH 2% + 100 mL NaClO and CP + 2%KOH + NaClO. Completely no growth of *C. gloeosporioides* was observed on the papyrus disc manufactured from strips pre-treated with PPWEx 2%, SLEx 2% and EuBEx 1% after 14 days from the incubation, compared to control treatments (Table 3). According to the visual observation, no growth of *A. niger* was found on the papyrus disc taken from the sheets treated with PPWEx 2%, Tyl 0.5% and Nano-cell + Tyl 0.5% (1:1 *v*/*v*). The inhibition zones (mm) as well as the growth on the discs are presented in Table 3.

Discs of Papyrus sheets produced from strips treated with extracts were found to have good antifungal activity, which is likely related to the presence of some active compounds. Therefore, Table 4 presents the suggested chemical composition of the extracts. Salem et al. [22] observed that wood specimens treated with 2% *P. rigida* heartwood extract had good inhibition against the growth of the following moulds: *Alternaria alternata*, *Fusarium subglutinans*, *Chaetomium globosum*, *A. niger* and *Trichoderma viride*.

Several chemical compounds, such as polyphenols, flavanoids, ellagitannis (tannins) and proanthocyanidins essential oils were found in *E. camaldulensis* extract [38]. These extracts have been shown to have good antifungal activities against certain molds [39,40]. Tritetracontane, octadecenoic acid-1,2,3-propanetriyl ester, hexadecanoic acid-methyl ester (10.5%) and 1,3-dioxane-4-(hexadecyloxy)-2-pentadecyl, as the main compounds, were identified on the leaf extract of *S. babylonica* [41]. The leaf extract of *S. babylonica* did not show any antibacterial and antifungal activity [42]. On the other hand, the aqueous extract of *S. babylonica* showed promising antifungal activity against *Fusarium oxysporum* [43]. Fungal growth, development and aflatoxin production by the fungus, *A. parasiticus*, were nearly eliminated by the application of bark volatile from *S. babylonica* [44]. *Phytophthora melonis* and *Pythium aphanidermatum*, the causal agents of cucumber root rot and damping, were not inhibited by the application of the leaf water extract of *S. babylonica* [45].

### 3.3. SEM Examination

Based on the visual observations of fungal growth, the papyrus sheet disc samples were chosen for SEM, as described in the material and methods section, while the other samples were not examined due to the inhibition zones shown in Table 3 or for the following reasons: 1. The samples were inhibited or fungal growth was prevented, 2. The samples were not inhibited but showed no fungal growth during the test period and 3. The samples showed weak fungal growth.

**Figure 8:**
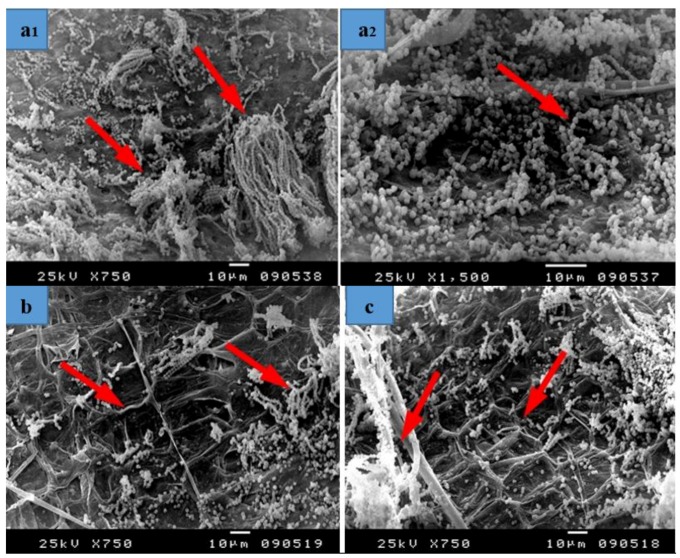

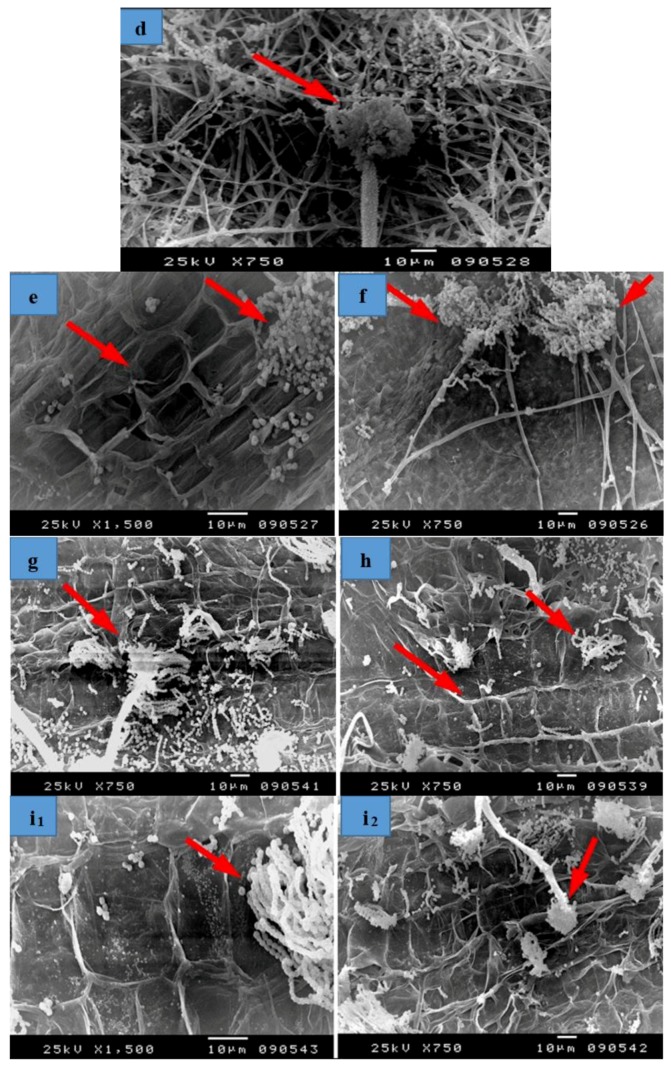
SEM images of papyrus sheets manufactured with some treatments and inoculated with *Aspergillus flavus*. (**a1**,**a2**) Papyrus sheet manufactured with strips treated with 10% DMSO; (**b**) Papyrus sheets manufactured with strips treated with *S. babylonica* leaf extract (2%); (**c**) Papyrus sheets manufactured with strips treated with *E. camaldulensis* bark extract (2%); (**d**) Papyrus sheet manufactured with strips soaked in tap water after hammering; (**e**) Papyrus sheets manufactured with strips treated with KOH (2%), then 100 mL NaClO for bleaching; (**f**) Papyrus sheets manufactured with strips treated with NaOH (2%), then 100 mL NaClO for bleaching; (**g**) Papyrus sheets manufactured with strips treated with nano-cellulose and tylose (1:1 *v*/*v*, 0.25%); (**h**) Papyrus sheets manufactured with strips treated with nano-cellulose and tylose (1:1 *v*/*v*, 0. 5%); (**i1**,**i2**) Papyrus sheets manufactured with strips treated with nano-cellulose (0.5%). Arrows refer to dense growth of the fungus.

SEM images of inoculated Papyrus sheets showed a huge growth of fungal mycelial of *A. flavus* (Figure 8) on strips treated with 10% DMSO (Figure 8a1,a2). A decrease in the fungal colonization as well as the appearance of cell walls were observed in the Papyrus strips pretreated with *S. babylonica* leaf extract (2%) (Figure 8b). Some growths of fungal mycelial as well as the appearance of Papyrus cell walls are shown in the strips pretreated with *E. camaldulensis* bark extract (2%) (Figure 8c). An intensive colonization growth of *A. flavus* is observed in the strips soaked in tap water after hammering (Figure 8d). A decrease in fungal hyphae growth and the appearance of erosion in the structure of papyrus cell walls is shown in strips pretreated with KOH (2%), then 100 mL NaClO (Figure 8e). Deterioration patterns are clearly shown in the cell structure of the strips pretreated with NaOH (2%), then 100 mL NaClO, with a decrease in fungal growth (Figure 8f). Some growths of fungal mycelial were shown in strips pretreated with nano-cellulose + tylose (1:1 *v*/*v*, 0.25%) (Figure 8g). A decrease in mycelial fungal growth was observed in strips pretreated with nano-cellulose and tylose (1:1 *v*/*v*, 0. 5%) (Figure 8h) and a decrease in the growth of fungal mycelial as well as cell wall consolidation was found in strips pretreated with nano-cellulose (0.5%) (Figure 8i1,i2).

**Figure 9:**
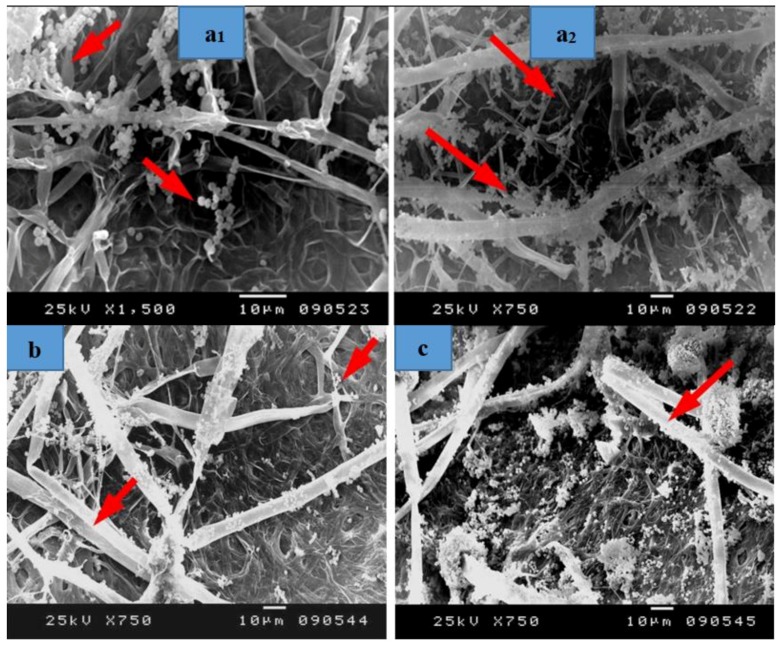

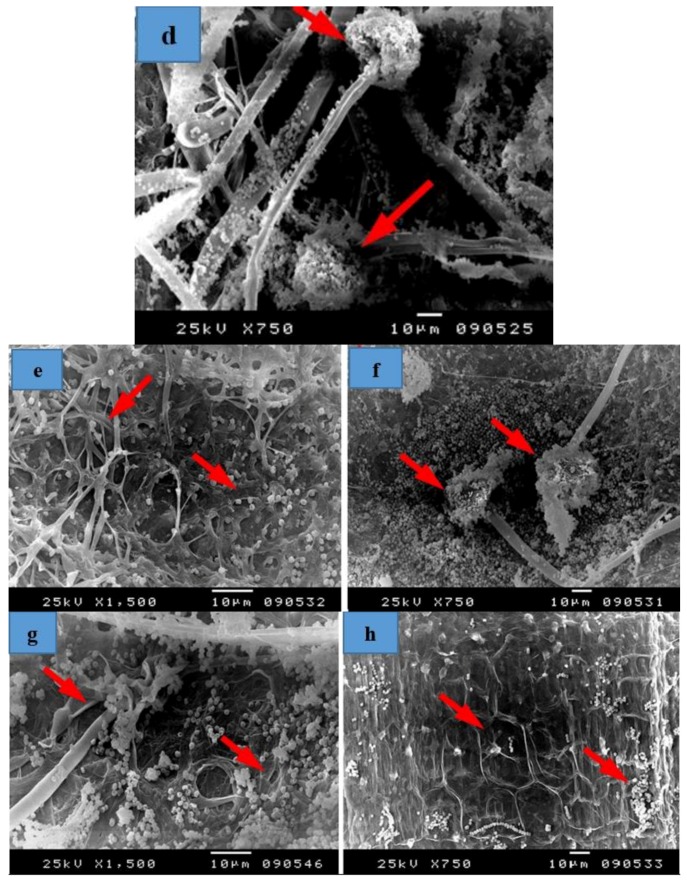
SEM images of papyrus sheets manufactured with some treatments and inoculated with *Aspergillus niger*. (**a1**,**a2**) Papyrus sheet manufactured with strips treated with 10% DMSO; (**b**) Papyrus sheets manufactured with strips treated with *S. babylonica* leaf extract (2%); (**c**) Papyrus sheets manufactured from strips treated with *E. camaldulensis* bark extract (2%); (**d**) Papyrus sheets manufactured with strips soaked in tap water after hammering; (**e**) Papyrus sheets manufactured with strips treated with KOH (2%), then 100 mL NaClO; (**f**) Papyrus sheets manufactured with strips treated with NaOH (2%), then 100 mL NaClO; (**g**) Papyrus sheets manufactured with strips treated with nano-cellulose and tylose (1:1 *v*/*v*, 0.25%); (**h**) Papyrus sheets manufactured with strips treated with nano-cellulose and tylose (1:1 *v*/*v*, 0. 5%). Arrows refer to dense growth of the fungus.

The Papyrus sample manufactured with strips treated with 10% DMSO showed a huge growth of fungal mycelial and inoculation with *A. niger* (Figure 9a1,a2). A decrease in the growth of fungal mycelial is shown in strips treated with *S. babylonica* leaf extract (2%) as well as the appearance of papyrus cells (Figure 9b). Some growth of fungal mycelial as well as the appearance of papyrus cell walls are shown in strips treated with *E. camaldulensis* bark extract (2%) (Figure 9c). A huge growth of fungal mycelial is observed in strips soaked in tap water after hammering (Figure 9d). Some growth of fungal mycelial and a change in cell structure was found in strips treated with KOH (2%), then 100 mL NaClO (Figure 9e). A huge growth of fungal mycelial is found in strips treated with NaOH (2%), then 100 mL NaClO (Figure 9f). Some growth of fungal mycelial is observed in strips treated with nano-cellulose and tylose (1:1 *v*/*v*, 0.25%) (Figure 9g). A decrease in the growth of fungal mycelial and cell walls, covered or consolidated, was found in strips treated with nano-cellulose and tylose (1:1 *v*/*v*, 0. 5%) (Figure 9h).

**Figure 10:**
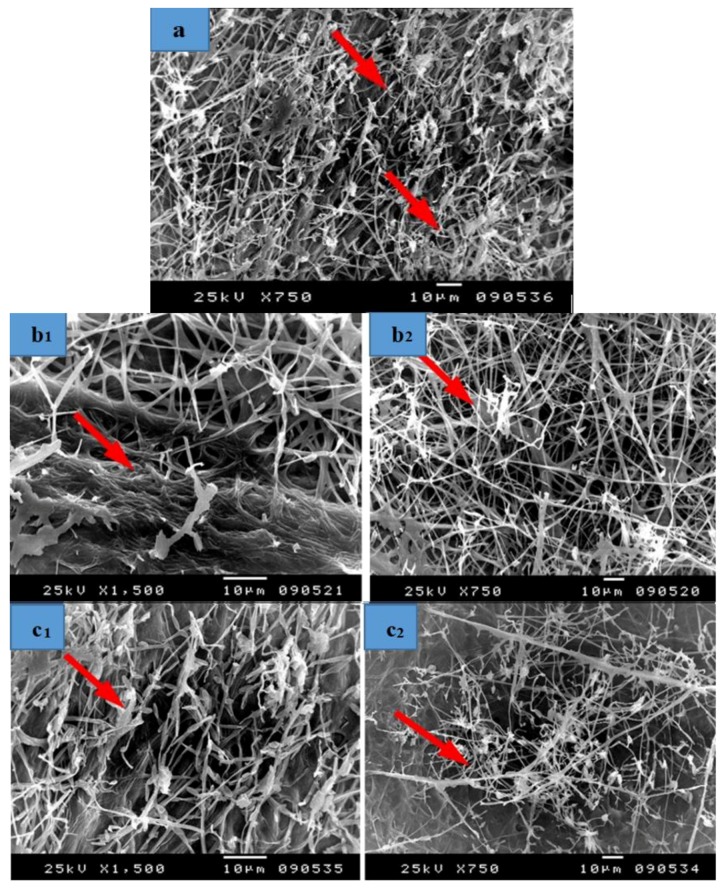

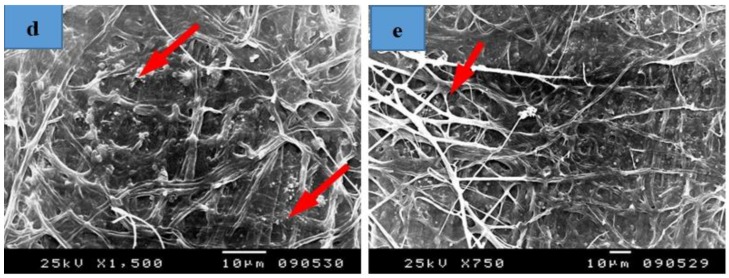
SEM images of Papyrus sheets manufactured with some treatments and inoculated with *Colletotrichum gloeosporioides*. (**a**) Papyrus sheets manufactured with strips treated with 10% DMSO; (**b1**,**b2**) Papyrus sheets manufactured with strips soaked in tap water; (**c1**,**c2**) Papyrus sheets manufactured with strips soaked in tap water and un-hammered; (**d**) Papyrus sheets manufactured with strips treated with KOH (2%), then 100 mL NaClO; (**e**) Papyrus sheets manufactured with strips treated with NaOH (2%), then 100 mL NaClO. Arrows refer to dense growth of the fungus.

The examined papyrus sheets inoculated with *Colletotrichum gloeosporioides* are presented in Figure 10. An intensive growth of fungal mycelial in strips treated with 10% DMSO (Figure 10a), those soaked in tap water (Figure 10b1,b2) and those soaked in tap water and un-hammered (Figure 10c1,c2) is shown. A decrease in the growth of fungal mycelial and a change in the structure of Papyrus cells is found in Papyrus strips treated with KOH (2%), then 100 mL NaClO (Figure 10d) and in those pretreated with NaOH (2%), then 100 mL NaClO (Figure 10e).

Nano-cellulose has been used as a consolidation material, for the improvement of the mechanical properties, as well as Tylose to enhance the fiber-fiber bond strength [46,47]. Some cellulose derivatives, such as Klucel G (Hydroxypropylcellulose), were found to have good consolidation properties to papyrus [6], with the best reduction in the growth of *A. nidulans*, *A. terrus*, *Penicillium asperum*, *Trichoderma viride* and *P. funiculosum* [48]. Sodium Carboxymethylcellulose (SCMC) was also used to consolidate the Papyrus [14].

## 4. Conclusions

This study evaluated the papyrus sheets produced from strips treated with different treatments. The mechanical properties of Papyrus strips were enhanced by pre-treating them with some individual or combination treatments. Strips treated with KOH or NaClO resulted in sheets with a high brightness, while other treatments caused sheets to have a darker colour. The natural extracts, with colouring chemical compounds (*P. rigida E. camaldulensis* and *S. babylonica*), that were applied to the strips were found to have good antifungal activities as well as to cause a reduction in the brightness of manufactured Papyrus sheets. Treatments, such as KOH 2% + 100 mL NaClO, CP + 2%KOH + NaClO, Tyl 0.5% and Nano-cell + Tyl 0.5% (1:1 *v*/*v*), showed no growth of *A. niger*. Deterioration or erosion patterns are clearly shown in papyrus cell structures in strips pre-treated with KOH or NaOH (2%), then chlorinated. Additionally, a huge growth of fungi was observed in strips pre-treated with DMSO-10% or soaked in tap water. Cell walls were consolidated with nano-cellulose or nano-cellulose with tylose treatments.

## Figures and Tables

**Table 1:** Different pre-treatments of Papyrus strips.

Treatment	Code	Strips Pre-Treatment	Abbreviation
T1	A	Treated with Pitch pine wood extract (2%)	PPWEx 2%
T2	B	Treated with Pitch pine wood extract (1%)	PPWEx 1%
T3	C	Treated with Pitch pine wood extract (0.5%)	PPWEx 0.5%
T4	D	Treated with *Salix babylonica* leaf extract (2%)	SLEx 2%
T5	E	Treated with S. *babylonica* leaf extract (1%)	SLEx 1%
T6	F	Treated with *S. babylonica* leaf extract (0.5%)	SLEx 0.5%
T7	G	Treated with *Eucalyptus camaldulensis* bark extract (2%)	EuBEx 2%
T8	H	Treated with *E. camaldulensis* bark extract (1%)	EuBEx 1%
T9	I	Treated with *E. camaldulensis* bark extract (0.5%)	EuBEx 0.5%
T10	J	Treated with 10% of dimethyl sulfoxide	DMSO 10% (control)
T11	K	Soaked in tap water after hammering	STWC (control)
T12	L	Treated with KOH (2%) + 100 mL NaClO for 1 h	KOH 2% + 100 mL NaClO
T13	M	Treated with NaOH (2%) + 100 mL NaClO for 1 h	NaOH (2%) + 100 mL NaClO
T14	N	Soaking in distilled water without hammering	SDW (control)
T15	O	Treated with tylose (0.5%) diluted in ethanol 95% and water	Tyl 0.5%
T16	P	Treated with tylose (0.25%) diluted in ethanol 95% and water	Tyl 0.25%
T17	Q	Treated with nano-cellulose 0.5% + Tylose 0.5% (1:1 *v*/*v*)	Nano-cell + Tyl 0.5% (1:1 *v*/*v*)
T18	q	Treated with mixture of nano-cellulose 0.25% + Tylose 0.25% (1:1 *v*/*v*)	Nano-cell + Tyl 0.25% (1:1 *v*/*v*)
T19	R	Treated with nano-cellulose (0.5%) dissolved in ethanol 95%	Nano-cell 0.5%
T20	S	Treated with nano-cellulose (0.25%) dissolved in ethanol 95%	Nano-cell 0.25%
T21	T	Soaked in tap water then chlorinated (100 mL NaClO) for 1 h	STW + 100 mL NaClO
T22	U	Hammered Papyrus sheets soaked in 2% KOH then chlorinated (100 mL NaClO) for 1 h and cold pressed	CP+2%KOH + 100 mL NaClO.
T23	V	Soaked un-hammered Papyrus sheets for 1 week in tap water then cold pressed without any treatments.	SUP (control)
T24	W	Commercial Papyrus sheets (hammered + KOH + Chlorination)	CPS (control)

**Table 2:** Effect of different strips’ pre-treatments on the mechanical and optical properties of manufactured papyrus sheets.

Strips Pre-Treatment	Tensile	Tear	Burst	Fold	Brightness	Darkness	Grammage
	N·m/g	mN·m^2^/g	kpa·m^2^/g	N	%	%	(g/m^2^)
PPWEx 2%	82.4 *^k^* ± 0.10	2123.33 *^h^* ± 0.57	568.33 *^n^* ± 0.58	3.66 *^defg^* ± 0.57	13.33 *^j^* ± 0.05	97.8 *^f^* ± 0.10	157.41 *^fg^* ± 6.41
PPWEx 1%	87.9 *^e^* ± 0.78	2232 *^c^* ± 1.73	785.33 *^f^* ± 1.53	4.33 *^cde^* ± 0.57	14.23 *^i^* ± 0.05	99.46 *^c^* ± 0.15	220 *^b^* ± 6.66
PPWEx 0.5%	83.4 *^j^* ± 0.20	2146.33 *^g^* ± 1.52	574 *^m^* ± 1	3.66 *^defg^* ± 0.57	14.2 *^i^* ± 0.1	99.76 *^ab^* ± 0.06	183.33 *^c^* ± 4.12
SLEx 2%	85.33 *^h^* ± 0.06	2197.33 *^e^* ± 1.15	683.33 *^i^* ± 3.05	4.33 *^cde^* ± 0.57	12.36 *^k^* ± 0.05	99.86 *^a^* ± 0.06	148.15 *^hi^* ± 3.21
SLEx 1%	84.6 *^i^* ± 0.10	2195.67 *^e^* ± 0.57	654.33 *^k^* ± 1.52	4 *^cdef^* ± 1	16.5 *^f^* ± 0.1	99.06 *^d^* ± 0.41	172.22 *^de^* ± 5.55
SLEx 0.5%	86.73 *^g^* ± 0.23	2212.33 *^d^* ± 0.57	774.33 *^g^* ± 0.57	4.66 *^cd^* ± 1.15	12.13 *^l^* ± 0.15	99.76 *^ab^* ± 0.06	165.43 *^ef^* ± 4.27
EuBEx 2%	81.33 *^l^* ± 0.06	2019 *^i^* ± 11.26	521 *^p^* ± 1	3 *^fgh^* ± 1	9.6 *^o^* ± 0.1	99.7 *^ab^* ± 0.10	157.57 *^fg^* ± 6.06
EuBEx 1%	88.66 *^d^* ± 0.06	1981.67 *^k^* ± 1.53	443 *^q^* ± 1	4 *^cdef^* ± 1	11.63 *^m^* ± 0.05	99.8 *^a^* ± 0.10	144 *^hi^* ± 0.00
EuBEx 0.5%	86.50 *^g^* ± 0.00	2174.33 *^f^* ± 11.01	647.67 *^l^* ± 0.57	5 *^c^* ± 1	11.53 *^m^* ± 0.05	99.86 *^a^* ± 0.06	141.56 *^i^* ± 2.85
DMSO 10% (control)	77.70 *^o^* ± 0.10	2343.67 *^a^* ± 0.57	787.33 *^e^* ± 1.15	3.33 *^efgh^* ± 0.57	11.56 *^m^* ± 0.05	98.76 *^e^* ± 0.06	128.21 *^j^* ± 3.17
STWC (control)	78.66 *^n^* ± 0.06	2234.33 *^c^* ± 0.57	666.33 *^j^* ± 1.15	4.33 *^cde^* ± 0.57	9.5 *^o^* ± 0.10	99.6 *^bc^* ± 0.10	191.14 *^c^* ± 4.03
KOH 2% + 100 mL NaClO	43.33 *^r^* ± 0.11	910.67 *^o^* ± 1.15	196.67 *^v^* ± 0.57	1.66 *^i^* ± 0.57	49.7 *^a^* ± 0.10	62.53 *^m^* ± 0.05	82.25 *^m^* ± 9.92
NaOH (2%) + 100 mL NaClO	55.16 *^q^* ± 0.21	871 *^p^* ± 1	312.67 *^u^* ± 1.15	2.33 *^hi^* ± 0.57	40.1 *^b^* ± 0.10	67.26 *^l^* ± 0.06	94.25 *^l^* ± 3.98
SDW (control)	80.23 *^m^* ± 0.06	1961.67 *^l^* ± 1.53	523.67 *^o^* ± 0.58	3.66 *^defg^* ± 0.57	14.3 *^i^* ± 0.26	97.66 *^f^* ± 0.11	174.59 *^d^* ± 0.40
Tyl 0.5%	87.56 *^f^* ± 0.06	2233 *^c^* ± 1	833.33 *^b^* ± 0.58	4.33 *^cde^* ± 0.57	13.36 *^j^* ± 0.15	95.56 *^h^* ± 0.06	368.88 *^a^* ± 7.69
Tyl 0.25%	89.76 *^c^* ± 0.06	2241.33 *^b^* ± 1.53	1162 *^a^* ± 1	4.66 *^cd^* ± 0.57	15.5 *^g^* ± 0.10	96.66 *^g^* ± 0.06	173.81 *^d^* ± 4.12
Nano-cell + Tyl 0.5% (1:1 *v*/*v*)	83.53 *^j^* ± 0.06	2211.67 *^d^* ± 1.53	795.33 *^d^* ± 0.58	4.66 *^cd^* ± 0.57	12.16 *^l^* ± 0.15	90.6 *^j^* ± 0.10	151.51 *^gh^* ± 6.06
Nano-cell + Tyl 0.25% (1:1 *v*/*v*)	83.46 *^j^* ± 0.06	2210.67 *^d^* ± 0.58	794.67 *^d^* ± 0.58	4.66 *^cd^* ± 0.57	12.1 *^l^* ± 0.10	90.6 *^j^* ± 0.10	129.29 *^j^* ± 3.49
Nano-cell 0.5%	98.66 *^a^* ± 0.06	2230.67 *^c^* ± 0.58	817 *^c^* ± 1	8.33 *^a^* ± 0.57	15.56 *^g^* ± 0.06	99.2 *^d^* ± 0.10	174.67 *^d^* ± 2.77
Nano-cell 0.25%	98.90 *^a^* ± 0.10	1873.67 *^m^* ± 1.53	723.33 *^h^* ± 0.58	3.66 *^defg^* ± 0.57	14.53 *^h^* ± 0.06	97.76 *^f^* ± 0.06	186.14 *^c^* ± 3.75
STW + NaClO	98.63 *^a^* ± 0.06	1587 *^n^* ± 1	320 *^t^* ± 1	7.33 *^ab^* ± 0.57	27.86 *^c^* ± 0.06	99.8 *^a^* ± 0.10	169.42 *^de^* ± 1.73
CP + 2%KOH + NaClO	93.26 *^b^* ± 0.23	1997 *^j^* ± 1	365.33 *^s^* ± 0.58	6.66 *^b^* ± 0.57	26.4 *^d^* ± 0.10	72.73 *^k^* ± 0.06	103.48 *^k^* ± 1.88
SUP (control)	89.73 *^c^* ± 0.06	1996.67 *^j^* ± 0.58	772.67 *^g^* ± 0.58	6.33 *^b^* ± 0.57	11.3 *^n^* ± 0.10	98.63 *^e^* ± 0.06	156.25 *^g^* ± 6.25
CPS (control)	60.40 *^p^* ± 0.10	807.67 *^q^* ± 0.58	388 *^r^* ± 1	2.66 *^ghi^* ± 0.57	25.43 *^e^* ± 0.06	95.13 *^i^* ± 0.11	151.85 *^gh^* ± 6.41
LSD_0.05_	0.325	5.557	1.794	1.144	0.175	0.196	8.08

Notes: Values are presented as mean ± SD; Means with the same letter within the same column are not significantly different according to LSD_0.05_.

**Table 3:** Screenings of the antifungal activity of produced Papyrus sheets against the growth of *A. niger*, *C. gloeosporioides* and *A. flavus*.

Treatment	Code	*A. flavus*				*C. gloeosporioides*				*A. niger*			
		Growth on Disc (mm)		Inhibition Zone (mm) *		Growth on Disc (mm)		Inhibition Zone (mm) *		Growth on Disc (mm)		Inhibition Zone (mm) *	
		7th day	14th day	7th day	14th day	7th day	14th day	7th day	14th day	7th day	14th day	7th day	14th day
PPWEx 2%	A	7–8	5–7	0	0	5–8	5–6	0	0	5–9	3–8	0	0
PPWEx 1%	B	2–4	1–2	0	0	3–5	0–2	0	1–2	0–1	0	1–5	5–8
PPWEx 0.5%	C	2–3	1–3	0	0	3–4	1–2	0	0–1	0–1	0	2–5	6–10
SLEx 2%	D	1–3	0–3	0	0–2	4–6	2–4	0	0	1–3	0–2	0–1	0–4
SLEx 1%	E	1–2	0–2	0	0–2	2–3	0–1	0	1–2	0	0	5–8	10
SLEx 0.5%	F	1–2	0–1	0–2	1–2	2–3	0–2	0	0	0	0	6–8	10
EuBEx 2%	G	2–3	1–2	0	0–1	2–4	1–3	0	0–1	1–2	0–1	0–2	1–5
EuBEx 1%	H	1–2	0–1	0–1	3–4	2–4	1–4	0	0	0	0	3–5	10
EuBEx 0.5%	I	0–1	0	1–2	5–7	1–2	0–2	0–1	1–3	0	0	4–7	10
DMSO 10%	J	0	0	2–5	8–10	0	0	0–1	3–8	0	0	5–7	10
STWC	K	0	0	1–2	9–10	0	0	0–2	3–9	0	0	3–5	10
KOH 2% +100 mL NaClO	L	0	0–1	0	0–1	0	0	0–1	1–2	0–5	0–2	0–1	1–2
NaOH (2%) + 100 mL NaClO	M	0	0	3–5	5	0	0	0–2	1–3	0	0	2–4	3–5
SDW	N	0	0	5–10	7–10	1-3	0	0–1	2–8	0	0	5–8	10
Tyl 0.5%	O	1–2	0	0–7	0–8	0	0	1–5	2–5	1–7	1–5	0	0
Tyl 0.25%	P	0	0	3–5	8–10	0	0	0–2	0–3	0–2	0–1	0–1	0–1
Nano-cell + Tyl 0.5% (1:1 *v*/*v*)	Q	0	0	3–6	6–8	0	0	0–1	0–1	1–4	1–3	0	0
Nano-cell + Tyl 0.25% (1:1 *v*/*v*)	q	0	0	10	10	0	0	0–2	0–2	0–2	0–1	0	0–2
Nano-cell 0.5%	R	0	0	5–8	7–10	0	0	1–2	1–2	0	0	1–2	1–4
Nano-cell 0.25%	S	0–3	0	0–6	2–8	0	0	1–3	2–3	0	0	0–2	1–5
STW + NaClO	T	0	0	0–3	1–9	0	0	0–1	1–2	0–5	0–1	0–1	0–2
CP + 2%KOH + NaClO	U	1–2	0	0	0–1	0	0	0	0–1	0–2	0	0–4	8–10
SUP	V	0	0	1–5	9–10	0	0	0–2	1–3	0–2	0–1	0–2	0–4
CPS	W	0	0	0–5	4–10	0	0	0–1	1–2	0–2	0	0–4	5–8

Notes: * Inhibition zones were recorded without adding the disc diameter. Each value in the table corresponds to the arithmetic mean of three treated papers, situated in three Petri dishes [27].

**Table 4:** Suggested chemical composition of extracts.

Extract	Main Chemical Compounds
*E. camaldulensis* bark extract	oleic acid (12.99%), oleic acid, hexyl ester (12.13%), 9-hexadecenoic acid (9.08%), 2-(acetyloxy)-1-[(acetyloxy)methyl]ethyl (9*E*,12*E*,15*E*)-9,12,15-octadecatrienoate (7.50%), (*Z*,*Z*)-9,12-octadecadienoic acid (5.79%), digitoxin (4.88%), 1,1-bis(dodecyloxy)hexadecane (3.50%), 9-octadecensaeure (3.36%), (*Z*,*Z*)-1,3-dioctadecenoyl glycerol (3.28%) and 2-(12-pentadecynyloxy)tetrahydro-2H-pyran (3.18%).
*P. rigida* heartwood *	*α*-terpineol (24.91%), borneol (10.95%), terpin hydrate (9.60%), D-fenchyl alcohol (5.99%), 2-pinen-4-ol (4.18%), 8-hydroxycarvotanacetone (2.62%), exo-2-hydroxycineole (2.45%), epoxylinalol (2.35%), oleic acid (2.29%) and carvone hydrate (2.09%).
*Salix babylonica* leaf extract	*Z*-8-methyl-9-tetradecenoic acid (8.74%), (*Z*)-9-octadecenoic acid (6.63%), 9-hexadecenoic acid (5.57%), 1,6-dihydrocarveol (3.35%), 3-[2-phenylethenyl]cholestan-2-one (2.26%), 2,6-dioxatricyclo[3.3.2.0(3,7)]decan-9-ol (2.06%), 9,12,15-octadecatrienoic acid, methyl ester (1.62%), 9,12-octadecadienoic acid (*Z*,*Z*)–(1.59%), 4α-phorbol 12,13-didecanoate (1.45%), tetrahydro-*α*,*α*,5-trimethyl-5-vinyl-furfuryl alcohol (1.24%), pentaneundecanoic acid (1.12%), 7-hydroxy-6-methyl-oct-3-enoic acid (1.03%), *E*-7-tetradecenol (1.01%), *Z*,*Z*,*Z*-1,4,6,9-nonadecatetraene (0.92%), (all-*Z*)-5,8,11,14-eicosatetraenoic acid, methyl ester (0.92%), 25-norisopropyl-9,19-cyclolanostan-22-en-24-one, 3-acetoxy-24-phenyl-4,4,14-trimethyl–(0.79%), (5*α*,17*β*)-androstan-3-one-17-methoxy-3-methoxime (0.63%) and 3-acetoxy-7,8-epoxylanostan-11-ol (0.40%).

Note: * Data from Salem et al. [22].
